# Gender Differences in Survival among Adult Patients Starting Antiretroviral Therapy in South Africa: A Multicentre Cohort Study

**DOI:** 10.1371/journal.pmed.1001304

**Published:** 2012-09-04

**Authors:** Morna Cornell, Michael Schomaker, Daniela Belen Garone, Janet Giddy, Christopher J. Hoffmann, Richard Lessells, Mhairi Maskew, Hans Prozesky, Robin Wood, Leigh F. Johnson, Matthias Egger, Andrew Boulle, Landon Myer, for the International Epidemiologic Databases to Evaluate AIDS Southern Africa (IeDEA-SA) Collaboration

**Affiliations:** 1 Centre for Infectious Disease Epidemiology & Research, School of Public Health & Family Medicine, University of Cape Town, Cape Town, South Africa; 2 Médecins sans Frontières, Cape Town, South Africa; 3 McCord Hospital, Durban, South Africa; 4 Aurum Institute, Johannesburg, South Africa and Johns Hopkins University School of Medicine; 5 Africa Centre for Health and Population Studies, University of KwaZulu-Natal, Mtubatuba, South Africa; 6 Health Economics & Epidemiology Research Office, Department of Internal Medicine, School of Clinical Medicine, Faculty of Health Sciences, University of the Witwatersrand, Johannesburg, South Africa; 7 Division of Infectious Diseases, Department of Medicine, University of Stellenbosch and Tygerberg Academic Hospital, Cape Town, South Africa; 8 The Desmond Tutu HIV Centre, Institute for Infectious Disease & Molecular Medicine, University of Cape Town, Cape Town, South Africa; 9 Division of International and Environmental Health, Institute of Social and Preventive Medicine (ISPM), University of Bern, Bern, Switzerland; Johns Hopkins University, United States of America

## Abstract

**Background:**

Increased mortality among men on antiretroviral therapy (ART) has been documented but remains poorly understood. We examined the magnitude of and risk factors for gender differences in mortality on ART.

**Methods and Findings:**

Analyses included 46,201 ART-naïve adults starting ART between January 2002 and December 2009 in eight ART programmes across South Africa (SA). Patients were followed from initiation of ART to outcome or analysis closure. The primary outcome was mortality; secondary outcomes were loss to follow-up (LTF), virologic suppression, and CD4+ cell count responses. Survival analyses were used to examine the hazard of death on ART by gender. Sensitivity analyses were limited to patients who were virologically suppressed and patients whose CD4+ cell count reached >200 cells/µl. We compared gender differences in mortality among HIV+ patients on ART with mortality in an age-standardised HIV-negative population.

Among 46,201 adults (65% female, median age 35 years), during 77,578 person-years of follow-up, men had lower median CD4+ cell counts than women (85 versus 110 cells/µl, *p*<0.001), were more likely to be classified WHO stage III/IV (86 versus 77%, *p*<0.001), and had higher mortality in crude (8.5 versus 5.7 deaths/100 person-years, *p*<0.001) and adjusted analyses (adjusted hazard ratio [AHR] 1.31, 95% CI 1.22–1.41). After 36 months on ART, men were more likely than women to be truly LTF (AHR 1.20, 95% CI 1.12–1.28) but not to die after LTF (AHR 1.04, 95% CI 0.86–1.25). Findings were consistent across all eight programmes. Virologic suppression was similar by gender; women had slightly better immunologic responses than men. Notably, the observed gender differences in mortality on ART were smaller than gender differences in age-standardised death rates in the HIV-negative South African population. Over time, non-HIV mortality appeared to account for an increasing proportion of observed mortality. The analysis was limited by missing data on baseline HIV disease characteristics, and we did not observe directly mortality in HIV-negative populations where the participating cohorts were located.

**Conclusions:**

HIV-infected men have higher mortality on ART than women in South African programmes, but these differences are only partly explained by more advanced HIV disease at the time of ART initiation, differential LTF and subsequent mortality, and differences in responses to treatment. The observed differences in mortality on ART may be best explained by background differences in mortality between men and women in the South African population unrelated to the HIV/AIDS epidemic.

*Please see later in the article for the Editors' Summary.*

## Abstract 2

Morna Cornell and colleagues investigate differences in mortality for HIV-positive men and women on antiretroviral therapy in South Africa.

## Editors' Summary

**Background:**

About 34 million people (most living in low- and middle-income countries) are currently infected with HIV, the virus that causes AIDS. HIV destroys CD4 lymphocytes and other immune system cells, leaving infected individuals susceptible to other infections. Early in the AIDS epidemic, most HIV-infected people died within 10 years of becoming infected. Then, in 1996, antiretroviral therapy (ART)—cocktails of drugs that keep HIV in check—became available. For people living in affluent countries, HIV/AIDS became a chronic condition. However, ART was expensive and, for people living in poorer countries, HIV/AIDS remained a fatal illness. In 2003, this situation was declared a global emergency, and governments and international agencies began to implement plans to increase ART coverage in resource-limited countries. Since then, ART programs in these countries have grown rapidly. In South Africa, for example, about 52% of the 3.14 million adults in need of ART were receiving an ART regimen recommended by the World Health Organization by the end of 2010.

**Why Was This Study Done?:**

The outcomes of ART programs in resource-limited countries need to be evaluated thoroughly so that these programs can be optimized. One area of concern to ART providers is that of gender differences in survival among patients receiving treatment. In sub-Saharan Africa, for example, men are more likely to die than women while receiving ART. This gender difference in mortality may arise because men initiating ART in many African ART programs have more advanced HIV disease than women (early ART initiation is associated with better outcomes than late initiation) or because men are more likely to be lost to follow-up than women (failure to continue treatment is associated with death). Other possible explanations for gender differentials in mortality on ART include gender differences in immunologic and virologic responses to treatment (increased numbers of immune system cells and reduced amounts of virus in the blood, respectively). In this multicenter cohort study, the researchers examine the size of, and risk factors for, gender differences in mortality on ART in South Africa by examining data collected from adults starting ART at International Epidemiologic Databases to Evaluate AIDS South Africa (IeDEA-SA) collaboration sites.

**What Did the Researchers Do and Find?:**

The researchers analyzed data collected from 46,201 ART-naïve adults who started ART between 2002 and 2009 in eight IeDEA-SA ART programs. At ART initiation, men had a lower CD4 count on average and were more likely to have advanced HIV disease than women. During the study, after allowing for factors likely to affect mortality such as HIV disease stage at initiation, men on ART had a 31% higher risk of dying than women. Men were more likely to be lost to follow-up than women, but men and women who were lost to follow-up were equally likely to die. Women had a slightly better immunological response to ART than men but virologic suppression was similar in both genders. Importantly, in analyses of mortality limited to individuals who were virologically suppressed at 12 months and to patients who had a good immunological response to ART, men still had a higher risk of death than women. However, the gender differences in mortality on ART were smaller than the gender differences in age-standardized mortality in the HIV-negative South African population.

**What Do These Findings Mean?:**

These analyses show that among South African patients initiating ART between 2002 and 2009, men were more likely to die than women but that this gender difference in mortality on ART cannot be completely explained by gender differences in baseline characteristics, loss to follow-up, or virologic and/or immunologic responses. Instead, the observed gender differences in mortality can best be explained by background gender differences in mortality in the whole South African population. Because substantial amounts of data were missing in this study (for example, HIV disease stage was not available for all the patients), these findings need to be interpreted cautiously. Moreover, similar studies need to be done in other settings to investigate whether they are generalizable to the South African national ART program and to other countries. If confirmed, however, these findings suggest that the root causes of gender differences in mortality on ART may be unrelated to HIV/AIDS or to the characteristics of ART programs.

**Additional Information:**

Please access these Web sites via the online version of this summary at http://dx.doi.org/10.1371/journal.pmed.1001304.

- Information is available from the US National Institute of Allergy and Infectious Diseases on HIV infection and AIDS
- Information on the treatment of HIV/AIDS in South Africa is available from the Southern African HIV Clinicians Society
- NAM/aidsmap provides basic information about HIV/AIDS and summaries of recent research findings on HIV care and treatment
- Information is available from Avert, an international AIDS charity on many aspects of HIV/AIDS, including information on HIV/AIDS treatment and care, and on HIV/AIDS in South Africa (in English and Spanish)
- WHO provides information about universal access to AIDS treatment (in several languages); its 2010 ART guidelines can be downloaded
- Information about the IeDEA-SA collaboration is available
- The Treatment Action Campaign provides information on antiretroviral therapy and South African HIV statistics
- Patient stories about living with HIV/AIDS are available through Avert; the nonprofit website Healthtalkonline also provides personal stories about living with HIV, including stories about taking anti-HIV drugs and the challenges of anti-HIV drugs

## Introduction

South Africa has the largest antiretroviral therapy (ART) programme worldwide. The programme has undergone rapid expansion with nearly 1.64 million adults initiating ART since 2004 [1]. Given the unprecedented scale of this initiative there is an urgent need to evaluate the outcomes of the programme in order to improve delivery of services. There is particular interest in gender differences in ART programme access and survival. Disproportionately more women than men have accessed ART in sub-Saharan Africa [2],[3]. Studies from Europe and North America suggest a higher risk of death on ART for women than men [4]; in contrast, across sub-Saharan Africa men appear to experience greater mortality than women on treatment [5]–[8].

A range of possible explanations for gender differences in mortality on ART have been suggested but there has been no comprehensive evaluation of the putative mechanisms. Baseline characteristics strongly predict mortality on ART [9]–[11], and men initiating ART in many African programmes have more advanced HIV disease than women [2],[6],[12]. In addition, loss to follow-up (LTF) is associated with mortality [13], and men are more likely to become LTF than women in many settings [14],[15]. Evidence regarding gender differences in immunologic and virologic responses is mixed [4]. It is vital to understand such differentials in order to improve health outcomes in this large and rapidly expanding health service.

We examined the magnitude of, and risk factors for, gender differences in mortality. We included data from eligible adults starting ART in the South African sites of the International Epidemiologic Databases to Evaluate AIDS Southern Africa (IeDEA-SA) collaboration between 2002 and 2009, representing about 10% of all patients enrolled nationally during this period [1]. We hypothesized that increased mortality in men on ART, if present, would be explained by differences in: (1) baseline characteristics; (2) differential risk of LTF and subsequent mortality; and/or (3) gender differences in virologic and immunologic responses.

## Methods

### Study Design, Population, and Eligibility Criteria

The South African cohorts of IeDEA-SA have been described in detail elsewhere [16],[17]. Briefly, the collaboration includes eight adult cohorts providing ART services in three of the most populous provinces (Gauteng, KwaZulu-Natal, and Western Cape). Cohorts range in size and are predominantly government-funded and follow national HIV treatment guidelines. The multicentre cohort is broadly representative of patients accessing public sector ART in rural and urban centres. This retrospective cohort analysis included all ART-naïve HIV-positive adults (≥16 and ≤80 y) who initiated ART between 2002 and 2009.

### Variables and Definitions

Baseline characteristics measured immediately before ART initiation included demographics (age, gender), available measures of HIV disease severity (CD4+ cell count, WHO stage, HIV viral load), clinical and laboratory characteristics (haemoglobin, weight), and calendar year of ART initiation. CD4+ cell count and viral load measures were taken after 12, 24, and 36 mo on ART. When measurements were not available at these time points, we included the closest laboratory measurement within a 3-mo period on either side of the date as available. All laboratory tests were performed by the South African National Health Laboratory Services.

We treated the following variables as categorical: age (16–24, 25–34, 35–44, 45+ y), CD4+ cell count (0–24, 25–49, 50–99, 100–199, 200+ cells/µl), WHO stage (I and II, III, IV), and haemoglobin; we treated weight and log viral load as continuous variables. Because of gender variability in haemoglobin levels, we generated a categorical variable (anaemia) from the haemoglobin level (measured in g/dl), which was defined as: none: females >11.9, males >13.1; mild: females 10–11.9, males 11–13.1; moderate: females 8.1 to <10, males 8.1 to <11; severe: <8.1 [18]. We defined virologic failure as a viral load measurement >400 copies/ml.

The primary outcome was mortality. Secondary outcomes were LTF, virologic suppression, and CD4+ cell count responses. Deaths were identified by the sites or by linkage to the National Population Register (NPR) of the Department of Home Affairs. Transfers were recorded by programmes and observation time was right-censored at the date of transfer. Patients were defined as LTF if there was no patient contact between analysis closure and database closure. Analysis closure preceded database closure by 6 mo to allow patients to meet the LTF definition. LTF date was defined as the last patient contact date. In order to differentiate between patients who were truly LTF and patients who had died within 3 mo of being LTF (misclassified deaths), we used linkage information to trace patients LTF with South African civil identification (ID) numbers (Figure S1). Patients who had a date of death within 3 mo after LTF were defined as misclassified deaths. Those with ID numbers who were not found in the population register in this period were defined “true” LTF [19]. For patients who started ART but had no further contact, we added 1 d of follow-up to allow their inclusion in survival analyses.

### Missing Data

On the basis of the assumption that data were likely missing at random, we used multiple imputation [20] by chained equation methods [21] to impute missing baseline data. We multiply imputed (20 times) baseline CD4+ cell count, WHO stage, viral load, weight, and haemoglobin. The multiple imputation models included all measured variables.

Given that a high proportion of patients LTF are likely to have died [13], we used inverse probability weighting [22] to correct mortality and LTF for missing deaths among those defined as LTF. Briefly, LTF patients with ID numbers (approximately 50%) were linked to the South African National Population Register to determine their true vital status (and date of death if deceased) and weighted to represent all patients LTF, enabling more accurate estimates of vital status.

### Analysis

Data were analysed using STATA 11.0 (STATA Corporation). Baseline characteristics were described with summary statistics (median, interquartile range [IQR] and proportions) by gender. Differences between proportions and medians were tested with Pearson's chi-squared test for proportions or the two-sample Wilcoxon rank-sum test. Two-sided statistical tests were used at alpha = 0.05. Time to death and time to “true” LTF were analysed from date of ART initiation using Kaplan-Meier curves.

Cox's proportional hazards regression models were used to assess crude and adjusted associations between patient characteristics and outcomes. All available plausible demographic and clinical variables were considered potential confounders and were included in multivariable models if they altered the association between gender and mortality or were significantly associated with the outcome under study. Results are presented as hazard ratios (HRs) with a 95% CI by duration on ART. The proportional hazards assumption was confirmed by testing gender/time and gender/log time interaction terms. We undertook sensitivity analyses limited to patients who were virologically suppressed and patients whose CD4+ cell count reached >200 cells/µl. We explored heterogeneity in analyses stratified by cohort. The gender mortality ratio was defined as the male divided by the female mortality rate.

To compare gender differences in our cohort with expected gender differences in the HIV-negative population, we calculated HIV-negative mortality in the South African male population and female population, age-standardised to our patients. Age-specific HIV-negative mortality rates in males and females were obtained from the Actuarial Society of South Africa (ASSA) estimates of non-HIV mortality in the year 2005 [23], which are derived from vital registration statistics as well as census and survey data (further explanation of the derivation of these rates is provided in Text S1).

All IeDEA-SA sites obtained ethical approval from relevant local institutions before contributing anonymised patient data to this collaborative analysis. In addition, the collaboration has approval from the University of Cape Town Human Research Ethics Committee to receive and analyse these collaborative data.

## Results

**Figure 1:**
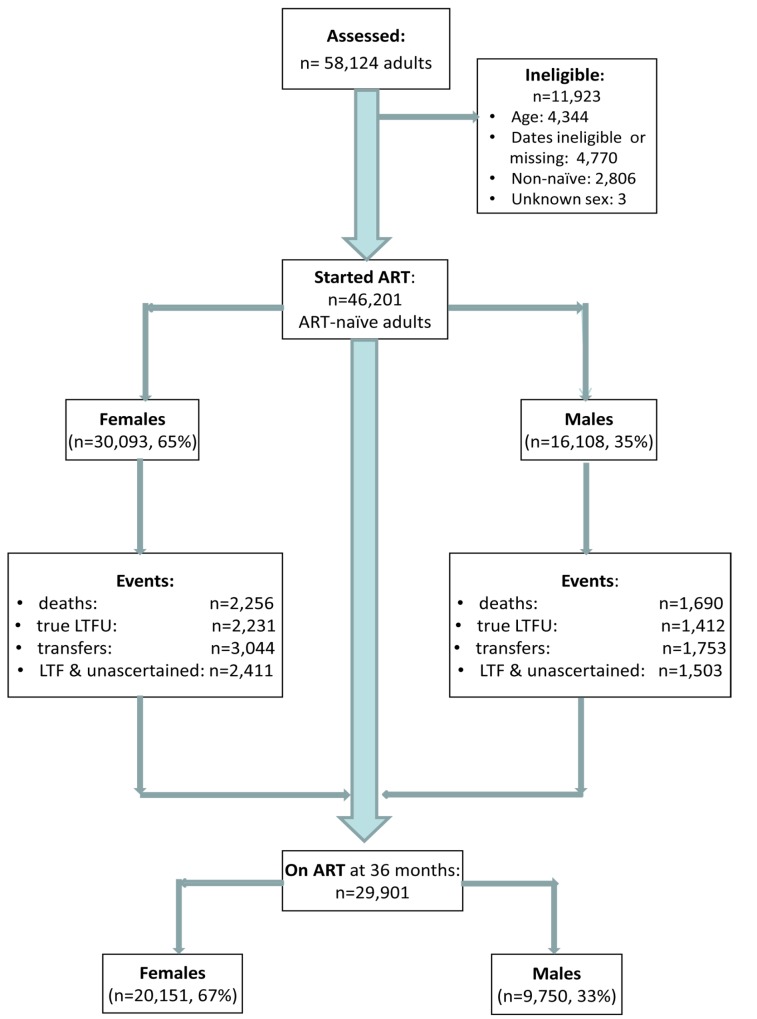
Patient flowchart. Description of a combined cohort of adult patients initiating public sector ART in South Africa, 2002–2009.

Among 58,124 patients assessed for eligibility, 11,923 were ineligible for the following reasons: age <16 or >80 y (*n* = 4,344), missing or invalid dates (birth, ART initiation, last visit, outcomes) (*n* = 4,770), non-ART naïve (*n* = 2,806), unknown gender (*n* = 3) (Figure 1). This analysis included 46,201 adults who started ART between 1 January 2002 and 31 December 2009 (median age 35 y; 65% female) (Table 1), contributing a total of 77,578 person-years of follow-up. Men had a shorter median time to death (483 versus 532 person-days) and “true” LTF (434 versus 495 person-days) than women. By the end of the study period, 29,901 patients were still on ART, 67% (*n* = 20,151) of these female.

**Table 1:** Patient characteristics among 46,201 adults initiating public-sector ART in South Africa, 2002–2009.

Baseline Characteristic^a^	Males (*n* = 16,108, 35%)	Females (*n* = 30,093, 65%)	Total (*n* = 46,201)
**Age,** median (IQR), years	37.5 (32.6–43.9)	33.4 (28.6–39.9)	34.9 (29.7–41.5)
16–24 *n* (%)^b^	442 (3)	3,124 (11)	3,566 (8)
25–34	5,639 (35)	14,214 (47)	19,853 (43)
35–44	6,486 (40)	8,810 (29)	15,296 (33)
≥45	3,541 (22)	3945 (13)	7,486 (16)
**CD4+ cell count**, median (IQR), cells/µl	85 (33–153)	110 (48–171)	101 (42–166)
0–24 *n* (%)	2,785 (20)	3,770 (15)	6,555 (16)
25–49	1,965 (14)	2,832 (11)	4,797 (12)
50–99	3,002 (22)	5,267 (20)	8,269 (21)
100–199	4,798 (35)	10,772 (41)	15,570 (39)
≥200	1,321 (10)	3,353 (13)	4,674 (12)
Missing data	14%	14%	14%
**WHO stage, ** ***n*** ** (%)**	5,538 (34)	11,290 (38)	16,828 (36)
I and II	790 (14)	2,704 (24)	3,494 (21)
III	3,221 (58)	5,933 (53)	9,154 (54)
IV	1,527 (28)	2,653 (24)	4,180 (25)
Missing data	66%	62%	64%
**Viral load, ** ***n*** ** (%)**	8,092 (50)	14,876 (49)	22,968 (50)
Log_10_ copies/ml, median (IQR)	4.9 (4.4–5.3)	4.8 (4.2–5.3)	4.8 (4.3–5.3)
Missing data	50%	51%	50%
**Haemoglobin, ** ***n*** ** (%)**	11,714 (73)	20,237 (67)	33,239 (72)
Median (IQR), g/dl	12 (10.2– 13.8)	10.9 (9.5–12)	11.1 (9.8–12.7)
Missing data	27%	33%	28%
**Anaemia, ** ***n*** ** (%)**	11,714 (73)	20,237 (67)	33,239 (72)
None	3,813 (33)	4,968 (23)	8,781 (26)
Mild	5,638 (48)	12,997 (60)	18,635 (56)
Moderate/severe	2,263 (19)	3,560 (17)	15,823 (18)
**Weight, ** ***n*** ** (%)**	12,740 (79)	23,788 (79)	36,528 (79)
Median (IQR), kg	60 (53.4–67.1)	59 (51–68)	59 (52–68)
**Pregnant, ** ***n*** ** (%)** c	—	1,494 (7%)	—
**Calendar year of ART initiation**			
2002 and 2003	439 (3)	856 (3)	1,310 (2.7)
2004	1,338 (8)	2,847 (9)	4,271 (8.6)
2005	2,743 (17)	5,568 (19)	8,640 (17.5)
2006	4,345 (27)	7,851 (26)	12,992 (26.3)
2007	3,629 (23)	6,506 (22)	10,944 (22.1)
2008	2,323 (14)	4,398 (15)	7,479 (15.1)
2009	1,291 (8)	2,067 (7)	3,836 (7.8)
**Follow-up,** median (IQR), person-days			
Time to death	483 (172–877)	532 (216–918)	515 (200–905)
Time to “true” LTF	434 (142–827)	495 (185–879)	476 (170–864)

a All differences between men and women were statistically significant (*p*<0.001).

b *n* (%) reflects the number (percentage) of patients with values for this variable.

c Data from five cohorts.

**Figure 2:**
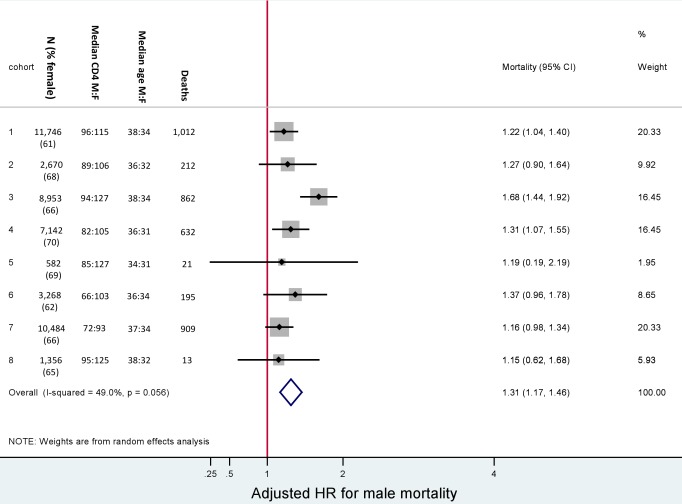
Male versus female mortality. Baseline characteristics and adjusted hazard ratios for male versus female mortality, by cohort. Adjusted for baseline age, CD4+ cell count, WHO stage, anaemia, weight, and viral load. Male versus female is reported as M∶F.

At initiation of ART, men were older than women (38 versus 33 y) and had lower median CD4+ cell counts (85 versus 110 cells/µl) (Table 1). Men were more likely than women to have a CD4+ cell count <50 cells/µl (34% versus 26%) and to be classified WHO stage III/IV (86% versus 77%). The median haemoglobin level was similar for men and women (12 versus 11 g/dl). Among females initiating ART, 7% were pregnant. Gender differences in baseline characteristics were consistent across all the eight cohorts (Figure 2).

### Gender and Mortality

**Figure 3:**
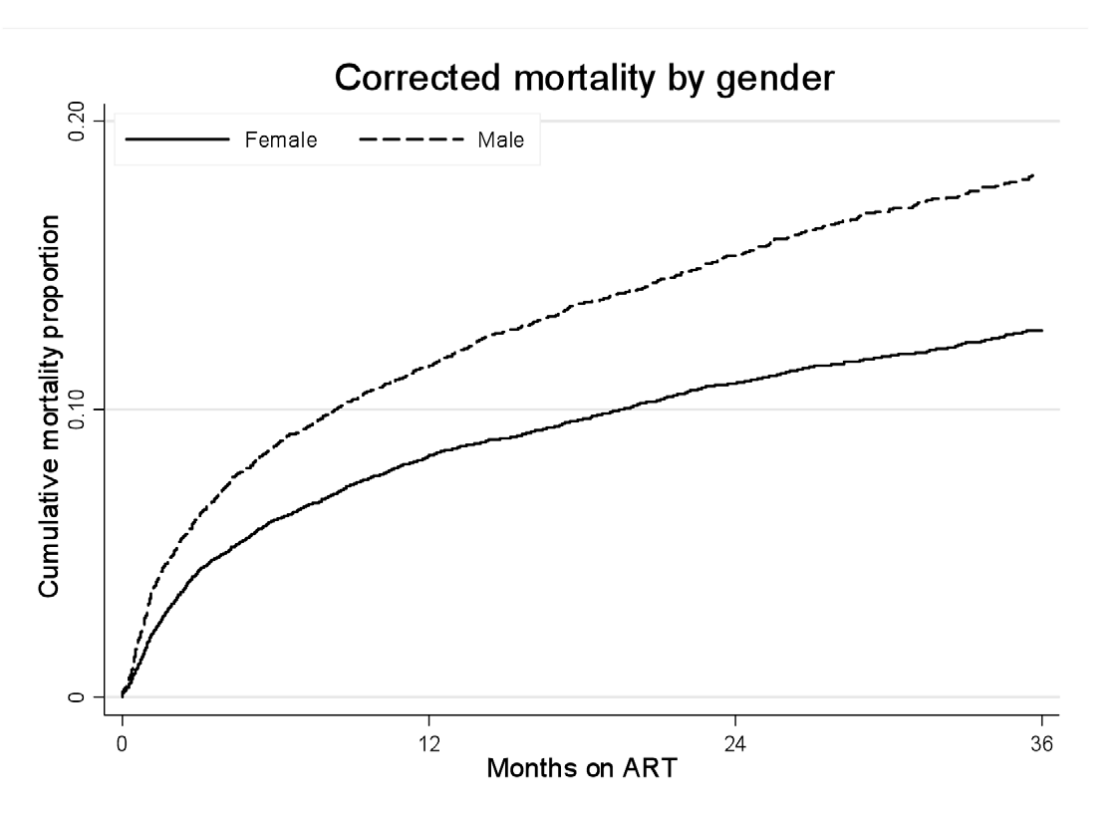
Mortality by gender and ART duration. Kaplan-Meier estimates of mortality by gender and duration on ART, corrected via linkage to the National Population Register.

In total, after correction via linkage to the National Population Register, there were 3,946 deaths, 57% among women. Men had a higher risk of mortality (Figure 3). The crude mortality on ART was higher for men than women: 8.5 versus 5.7/100 person-years, unadjusted HR 1.46 (1.37–1.56), *p*<0.001. In multivariable analysis, after adjusting for baseline age, cohort, CD4+ cell count, WHO stage, log viral load, anaemia, and weight, men had a 31% higher risk of death than women (adjusted HR [AHR] 1.31, 95% CI 1.22–1.41) (Table 2). Other baseline factors associated with mortality were age >35 y, CD4+ cell count, WHO stage, anaemia, weight, and viral load (Table S1). The association between gender and death persisted with increasing duration on ART. In a stratified analysis, the elevated risk of death for men compared with women was consistent across cohorts (Figure 2). Excluding WHO stage from the adjusted model did not change our main finding (AHR 1.34, 95% CI 1.25–1.44). There was no evidence of an interaction between gender and age.

**Table 2:** Crude and adjusted associations between male gender and mortality by duration on ART.

Duration	Crude HR	AHR
**0–12 mo**	1.28 (1.09–1.51)	1.10 (0.93–1.31)
**12–24 mo**	1.63 (1.37–1.94)	1.36 (1.05–1.78)
**24–36 mo**	1.62 (1.22–2.14)	1.39 (0.94–2.06)
**>36 mo**	1.71 (1.08–2.70)	1.35 (0.76–2.38)
**Total time**	1.46 (1.37–1.56)	1.31 (1.22–1.41)

Multivariable models adjusted for cohort, age, CD4+ cell count, WHO stage, anaemia, weight, and log viral load at ART initiation. 95% CI in brackets after HR and AHR.

### Gender and Loss to Follow-up

Using our initial definition, 8,303 adults were suspected LTF, of whom 61% were female (Figure S1). We were able to ascertain the vital status of 4,389 patients who had ID numbers (61% female). Among those patients who were LTF without ID numbers and whose outcomes could not be ascertained (*n* = 3,914), 62% were female. After linkage to the NPR, we identified 746 patients who had died within 3 mo of being suspected LTF. We regarded these as misclassified deaths (57% female). After correction for these deaths, a total of 3,643 patients were defined as “true” LTF, 61% female.

**Figure 4:**
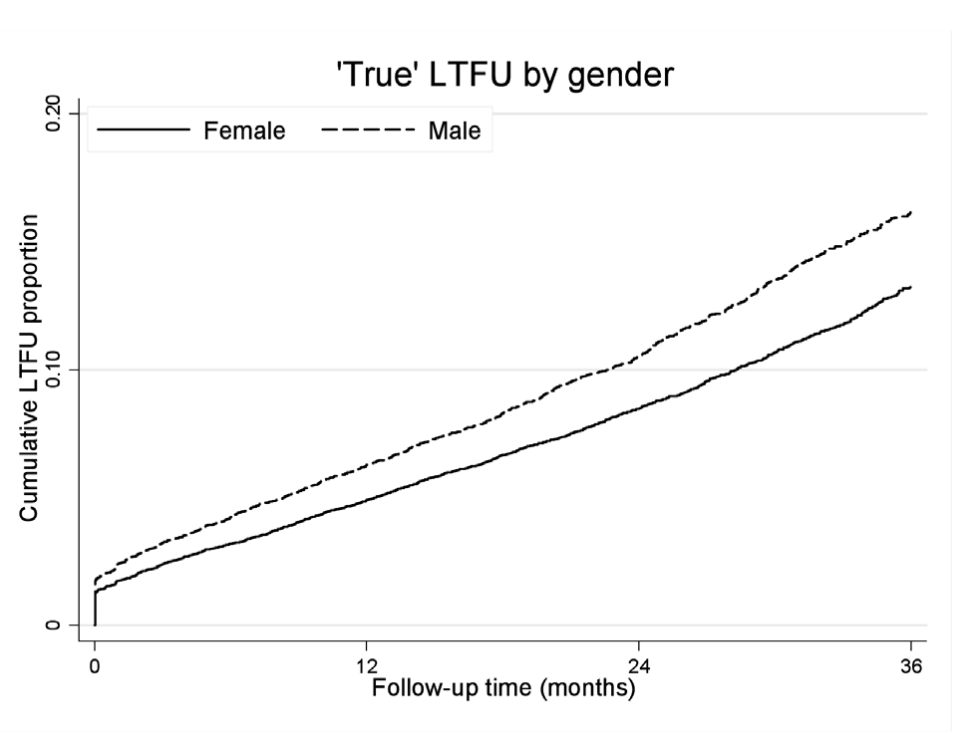
“True” loss to follow-up by gender and ART duration. Kaplan-Meier estimates of “true” LTF by gender and duration on ART.

Men were more likely than women to experience “true” LTF (Figure 4). The crude “true” LTF rate was 10.03/100 person-years, lower among women than men (9.18 versus 11.77/100 person-years, respectively). In multivariable analysis, after adjusting for baseline age, cohort, CD4+ cell count, WHO stage, anaemia, weight, viral load, and calendar year of initiation, men were more likely to be truly LTF than women (AHR 1.20, 95% CI 1.12–1.28). Within each cohort, the trend towards increased risk of “true” LTF was consistent but estimates were less precise. Using linkage to the NPR, we then explored mortality among those who were truly LTF with ID numbers (i.e., still alive 3 mo after being suspected LTF). We found no gender difference in the hazard of death among those patients who were truly LTF (AHR 1.04, 95% CI 0.86–1.25).

### Gender and Virologic and Immunologic Responses to ART

There was a high proportion of virologically suppressed patients at 12, 24, and 36 mo on ART, with no evident gender difference in proportions (Table 3). When analysis of mortality was restricted to individuals who were virologically suppressed at 12 mo, men still had a higher risk of death than women (AHR 1.38, 95% CI 1.07–1.79). Women initiating ART had a higher baseline CD4+ cell count and better CD4+ cell count responses than men over 36 mo (Figure S2). The median incremental CD4+ cell gains for women and men at 12, 24, and 36 mo were 179 versus 145, 89 versus 70, and 63 versus 39 cells/µl, respectively (Table 3, *p*<0.001 for all comparisons). In a sensitivity analysis limited to patients who had reached a CD4+ cell count ≥200 after a year on ART, the gender difference in mortality persisted (AHR 1.37, 95% CI 1.03–1.83) (unpublished data).

**Table 3:** Virologic and immunologic responses by gender and duration on ART.

Measurement	12 mo		24 mo		36 mo	
	Female (n = 21,032)	Male (n = 10,702)	Female (n = 13,232)	Male (n = 6,826)	Female (n = 7,440)	Male (n = 3,950)
**Viral load tests**						
Total tested, n	13,529	6,600	8,280	3,798	4,225	1,958
Proportion suppressed^a^	0.87	0.86	0.86	0.86	0.93	0.95
Proportion missing data	0.36	0.38	0.37	0.44	0.43	0.50
**CD4+ cell counts**						
Total tested, n	15,882	7,752	9,510	4,438	4,728	2,168
Median, cells/µl	294	239	381	315	437	359
(IQR)	(204 to 403)	(164 to 337)	(268 to 512)	(217 to 431)	(313 to 582)	(243 to 493)
Median CD4+ cell count increase^b^	179	145	89	70	63	39
(IQR)	(100 to 273)	(78 to 223)	(13 to 175)	(9 to 141)	(−22 to 147)	(−21 to 114)
Proportion missing data	0.24	0.28	0.28	0.35	0.36	0.45

a Proportion suppressed is the proportion of patients with available data who achieved a viral load ≤400 copies/ml.

b Median CD4+ cell count increase is the difference in CD4+ cell count at each time point between this measure and the measure taken at the previous time point; all differences between men and women were statistically significant, *p*<0.001.

### Gender and Non-HIV Mortality

**Figure 5:**
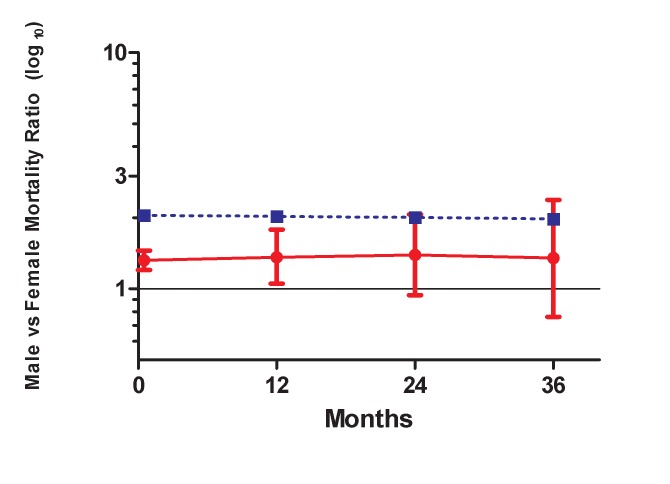
Male versus female mortality ratios over time. Solid line, observed mortality ratio for men versus women on ART. Dotted line, age-standardised HIV-negative mortality ratio for men versus women in South Africa.

Given the persistence of the gender differential in mortality among patients on ART, we compared this to the background gender differential in mortality in the South African population. Figure 5 shows that the gender mortality ratio among patients on ART appears to be smaller than the age-standardised HIV-negative mortality ratio for men versus women in South Africa. HIV-negative men with the same age distribution as that in the ART cohort would be expected to die at twice the rate in HIV-negative women of the same age, compared with our AHR of 1.31 on ART. With increasing duration of ART, the contribution of expected non-HIV mortality to observed mortality increased from an estimated 5% in the first 6 mo to 36% after 36 mo among men and from 3% to 25% among women during the same periods (Table 4). In further analyses stratified by age (16–34 and 35+ y), expected non-HIV mortality accounted for a larger proportion of deaths in the older men than in the younger men (Table S2). One exception was the proportion of observed male mortality attributable to non-HIV mortality at >36 mo, likely to be random error due to the small number of deaths at the longest duration. In both age groups, non-HIV mortality contributed more substantially to male mortality than to female mortality. In addition, over all periods on ART, although the expected ratio of male to female non-HIV deaths differed at younger and older ages, all ratios were greater than the male to female ratio of observed mortality on ART.

**Table 4:** Observed crude mortality^a^, age-standardised HIV-negative mortality^a^, and expected non-HIV mortality as a percentage of observed mortality among men and women, by duration on ART.

Gender	Mortality	Duration on ART				
		0–6 mo	6–12 mo	12–24 mo	24–36 mo	>36 mo
**Male**	Observed mortality (crude)	19.13	6.19	4.52	3.43	2.86
	HIV-negative mortality (age-standardized)	0.90	0.92	0.95	0.98	1.03
	Expected non-HIV mortality as a percentage of observed mortality	5%	15%	21%	29%	36%
**Female**	Observed mortality (crude)	13.16	4.82	2.78	2.12	1.69
	HIV-negative mortality (age-standardized)	0.36	0.37	0.38	0.39	0.42
	Expected non-HIV mortality as a percentage of observed mortality	3%	8%	14%	19%	25%

## Discussion

These analyses demonstrate that among patients initiating ART between 2002 and 2009 at sites across South Africa, men had higher mortality than women. We hypothesized that gender differences in mortality would be explained by differences in baseline characteristics, LTF and subsequent mortality, and/or virologic and immunologic responses. However, we found that the increased mortality risk among men persisted throughout each analysis, including after adjustment for measures of HIV disease at the time of ART initiation, in the subset of patients who achieved virologic suppression, and among patients with good immune responses to treatment.

There is a large and growing body of work on gender and ART in developing countries, much of which documents the same association repeatedly [24]: disproportionately fewer men than women access ART [2],[3],[25], and there is higher mortality among men than women on ART [5],[8],[15],[16],[26],[27]. Such studies typically speculate as to the possible mechanisms underlying the observed associations. Putative mechanisms include: poor health-seeking behaviours among men leading to more advanced disease at the time of ART initiation, differential rates of LTF leading to higher mortality, behavioural factors such as poor adherence, and/or biologic factors such as gender differences in immunologic responses to ART. However, there have been few systematic attempts to evaluate each of these possible mechanisms.

In this study, women comprised the majority of adults starting ART across the country. In the early years of ART programmes there were understandable concerns that due to gender imbalances, women may have reduced access to ART services [28]. Contrary to this, however, there is mounting evidence that men appear disadvantaged in their access to ART programmes. Numerous papers and a systematic review have suggested gender inequalities in ART access, particularly in sub-Saharan Africa [2],[3],[8], and ART initiation in South Africa, relative to ART need, is substantially higher in women than in men [1].

Among patients who do start ART, late presentation has been cited as one of the main reasons for increased male mortality in ART programmes [4]–[6],[8]. In sub-Saharan Africa, men appear to initiate ART at older ages and with more advanced HIV disease than women [2],[3],[6],[16], and markers of advanced HIV disease at the time of ART initiation strongly predict early mortality on ART [9],[12],[29]–[31]. However, in our multivariable models we found that adjustment for baseline characteristics accounted for only part of the gender difference in mortality, and that the gender differentials in mortality continued to appear for several years after ART initiation. The reasons for men's later entry into ART programmes are poorly understood but frequently are attributed to gender differences in health-seeking behaviour or in routes of referral [32]. An alternate and more compelling explanation may be that while prioritising maternal and child health services in many public health systems, men's primary health care needs may have been neglected [32]–[35], a possibility that warrants further research attention.

We then assessed the role of LTF in explaining mortality differentials. Increased mortality among patients LTF, especially within the first 3 mo after LTF, has been well documented [19],[36]–[40]. In turn, it is plausible that if males are more likely to be LTF, this could explain their elevated mortality risk. Through linkage to the South African Population Register we were able to identify a group of patients who were truly LTF, and to trace their vital status after being LTF. Although men were more likely to be LTF compared with women, we found no gender difference in mortality after LTF, and deaths after LTF contributed only a small proportion of all deaths. Thus in these data, LTF alone did not appear to explain men's increased mortality.

Behavioural factors such as treatment adherence could also help to explain the observed increased male mortality on ART. Poor adherence to ART, measured by virologic non-suppression, significantly increases the risk of mortality on treatment. We found no apparent gender difference in virologic suppression, although previous studies have suggested that adherence may vary by gender [41]. Of note, this finding was consistent across all cohorts, and in a sensitivity analysis restricted to patients who were virologically suppressed at 12 mo, men still had a higher risk of subsequent death than women. As a result, gender differences in adherence to treatment do not appear to explain differences in mortality.

Biologic differences between men and women have been suggested as shaping immunologic responses to ART and mortality risk. We found that women had higher CD4+ cell counts at ART initiation than men, and slightly better absolute CD4+ cell increases on treatment. These data are in line with results from two collaborative studies in sub-Saharan Africa that documented greater immune recovery in women than in men, with gender-based differences increasing with time on ART [43],[44]. However, even among patients with similar immunologic responses by 1 y on treatment, the gender difference in mortality persisted.

In these data, the finding of persistently increased male mortality for up to 3 y on ART does not appear to be explained entirely by baseline differences in HIV disease status, variation in LTF, differences in virologic suppression, or sex-linked differences in immune responses to treatment. Having thus refuted our a priori hypotheses, we explored alternate explanations for the observed association. We examined evidence for gender differences in non-HIV adult mortality in South Africa, and found pronounced differences that appear independent of the HIV/AIDS epidemic. Specifically, evidence from actuarial modelling suggests that HIV-negative South African men in the same age groups as our study population have twice the mortality risk of women, although the male-female differences tend to be more pronounced in young adults than in older adults. This pattern is not unique to South Africa: similar trends are seen elsewhere in Africa and worldwide [44]–[48]. This evidence places these data, and previous studies showing increased mortality among men in ART services across Africa, into perspective: among patients on ART, men have an increased risk of death on ART compared to women, but this same phenomenon operates in HIV-negative individuals. In fact, in South Africa the mortality ratio for men versus women on ART appears to be somewhat smaller than the age-standardised ratio for men versus women estimated for the HIV-negative population. Gender differentials in mortality of HIV-negative individuals are well-documented in South Africa and include an increased burden of mortality among younger men due to traumatic causes and non-HIV tuberculosis [49]. It is possible that through accessing ART services, men and women may access other preventive and curative services that reduce non-HIV mortality. While these findings are intriguing, it is important to recognize that our original hypotheses did not include gender differences in non-HIV mortality, and this phenomenon clearly requires further investigation.

Observational studies of patients on ART face major constraints in terms of mortality ascertainment, LTF, and missing measurements. Most cohort analyses from sub-Saharan Africa—and especially large collaborative analyses—have limited ability to ascertain true outcomes as they have little capacity to follow patients actively [6]. In addition, few African countries have high quality vital statistics [47],[50],[51]. In turn, most large ART programmes have difficulty confirming patients' true vital status [52], and in the absence of reliable death registration, researchers have undertaken targeted tracing studies [40]. In South Africa, the National Population Register captures nearly 90% of adult deaths [53]. Through our linkage with this register, we are able to distinguish between “true” LTF and unrecognised mortality in many patients. Moreover, consistency of findings across different cohorts strengthens the generalizability of our results.

Interpretation of these results is subject to several important limitations. These data come from multiple service delivery programmes from across South Africa, and there is a substantial amount of missing data in this analysis. For example, South African national identity numbers were only available for 53% of those who were suspected to be LTF, and it is possible that there are systematic differences between patients with and without ID numbers for which we have not accounted. There was also a high proportion of missing values for WHO stage and viral load; we tried to address this with multiple imputation and sensitivity analyses confirming our main findings. Missing data on specific risk factors for mortality, particularly prevalent tuberculosis at ART initiation [54], further limited this analysis. Finally, we did not observe mortality in HIV-negative populations where the participating cohorts are located, and instead used a national-level actuarial model to estimate mortality in men and women who are HIV-negative. These findings are likely to be generalizable to the South African national ART programme, and potentially to other parts of the region, but further investigation in other settings is warranted.

In summary, there have been concerns raised about the presence of gender differences in mortality on ART in South Africa and many other parts of the continent. In this study we systematically explored this phenomenon and found that none of the explanations posited for this association adequately explains the increased mortality observed among men on ART. Instead, the observed differences in mortality on ART may be best explained by background differences in death rates between men and women in the South African population, unrelated to the HIV/AIDS epidemic.

## Supporting Information

Figure S1 **Determining “true” loss to follow-up.**
(TIF)

Figure S2 **Crude CD4+ cell count responses by gender, 0–36 mo on ART.**
(TIF)

Table S1 **Crude and adjusted associations between male gender and mortality.**
(TIF)

Table S2 **Mortality by duration on ART, stratified by age.**
(TIF)

Text S1 **Method for estimating non-HIV mortality.**
(DOCX)

## Abbreviations

AHR: adjusted hazard ratio
ART: antiretroviral therapy
HR: hazard ratio
ID: identification
IQR: interquartile range
LTF: loss to follow-up
