# Supplementary figures and images for: Gender Differences in Survival among Adult Patients Starting Antiretroviral Therapy in South Africa: A Multicentre Cohort Study
Source: PLoS Med. 2012 Sep 4;9(9):e1001304. doi: 10.1371/journal.pmed.1001304 (PMC3433409; doi:10.1371/journal.pmed.1001304)

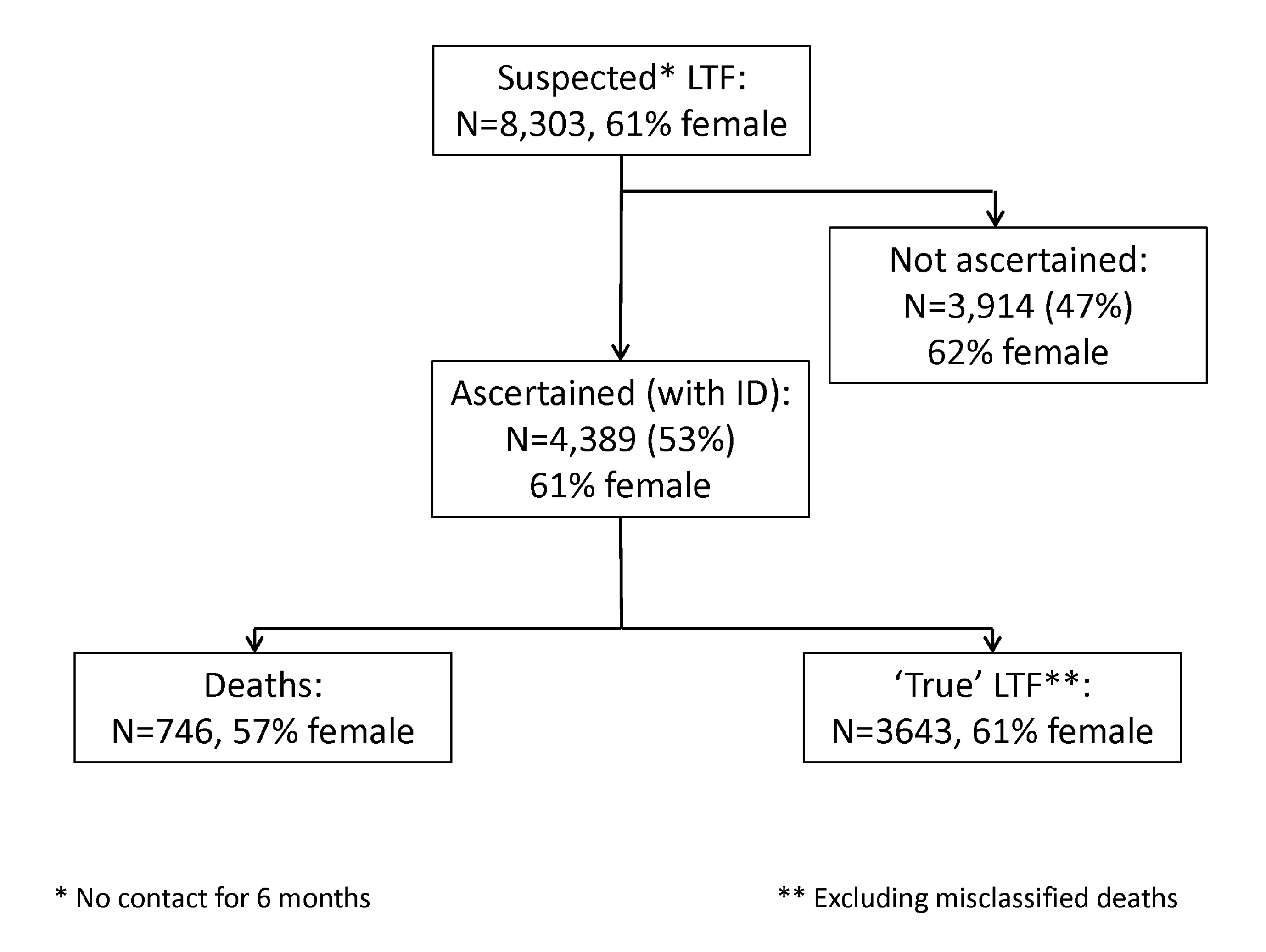

Supplement: Figure S1 — Determining “true” loss to follow-up. (TIF) [file pmed.1001304.s001.tif]

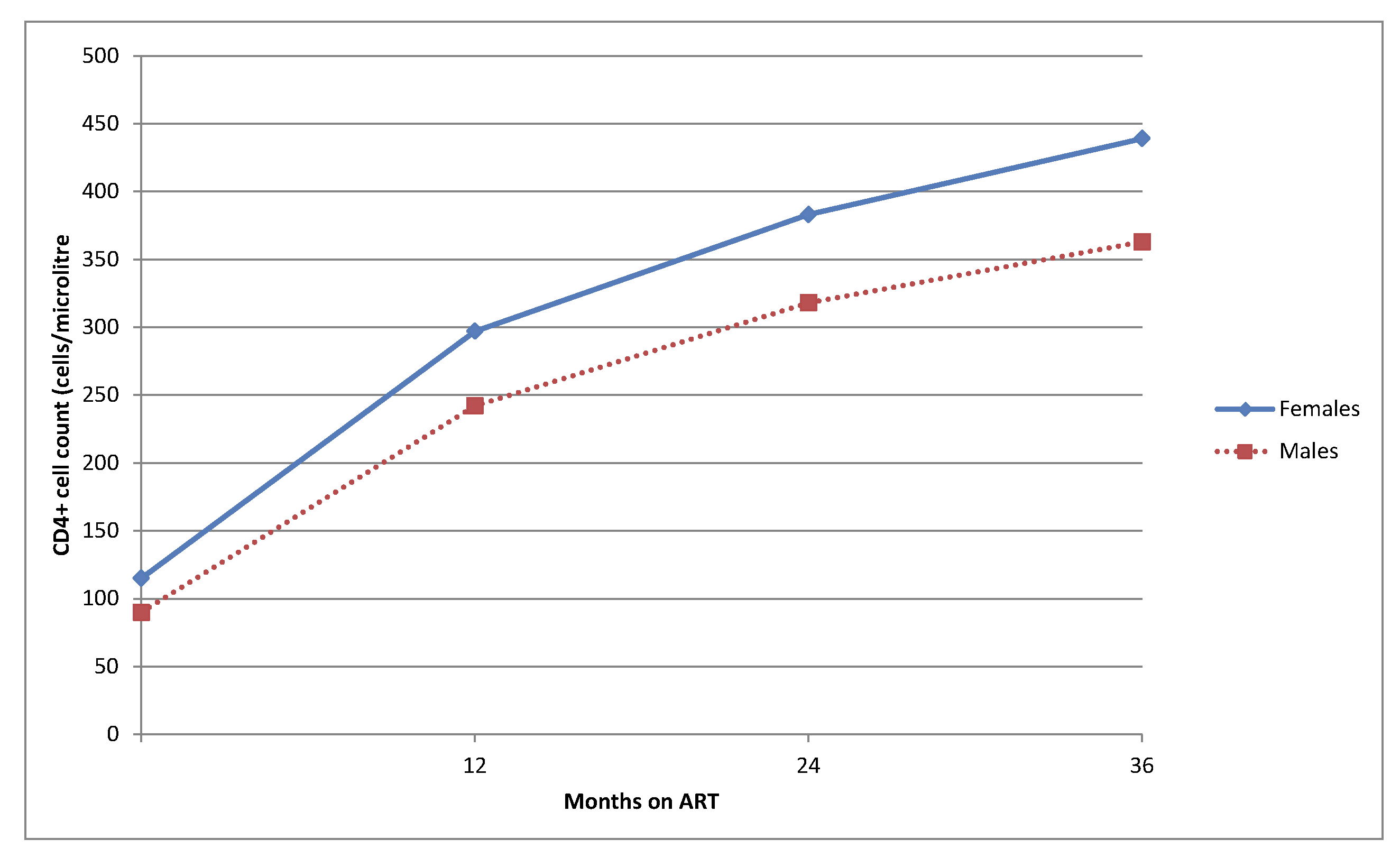

Supplement: Figure S2 — Crude CD4+ cell count responses by gender, 0–36 mo on ART. (TIF) [file pmed.1001304.s002.tif]

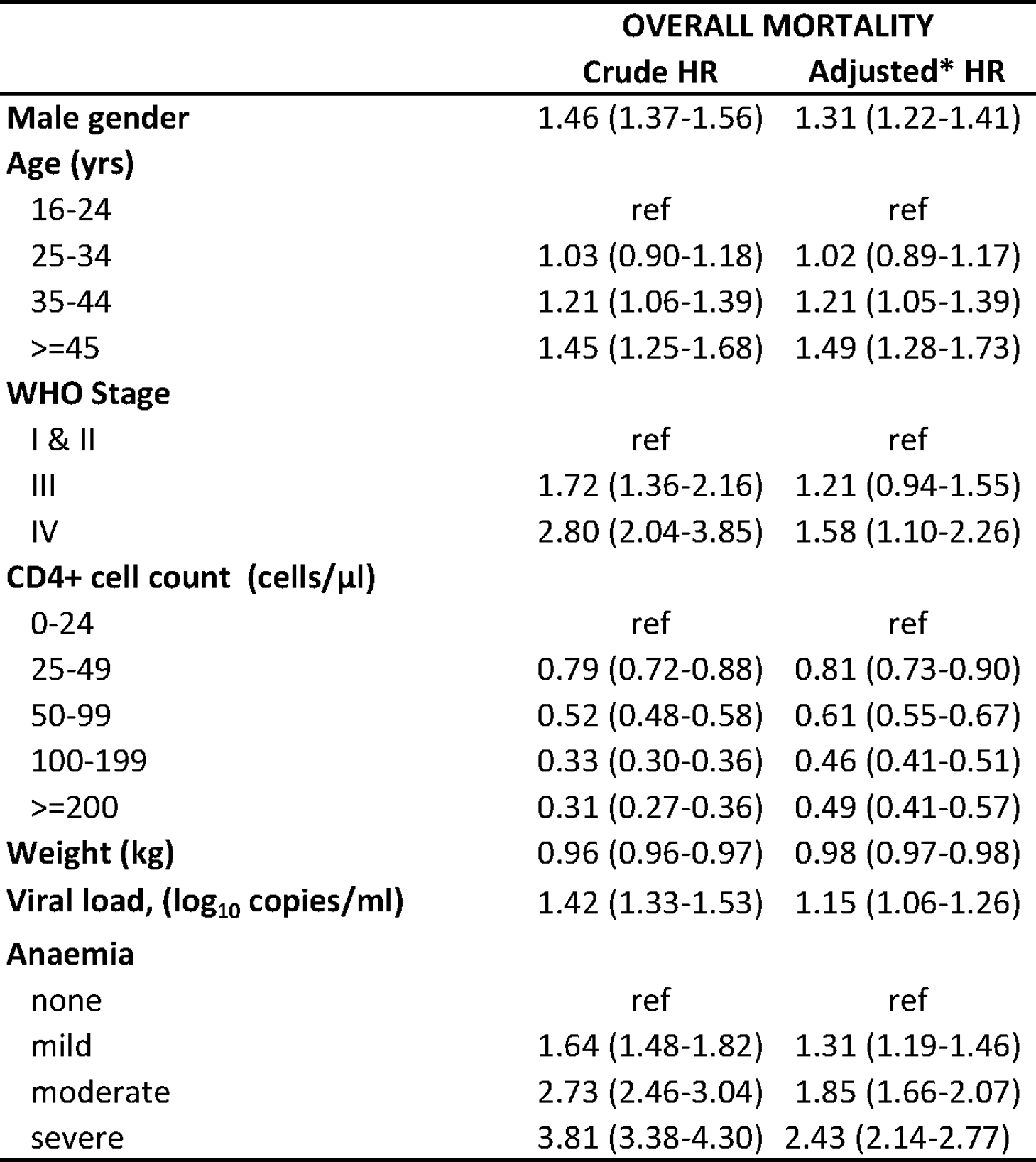

Supplement: Table S1 — Crude and adjusted associations between male gender and mortality. (TIF) [file pmed.1001304.s003.tif]

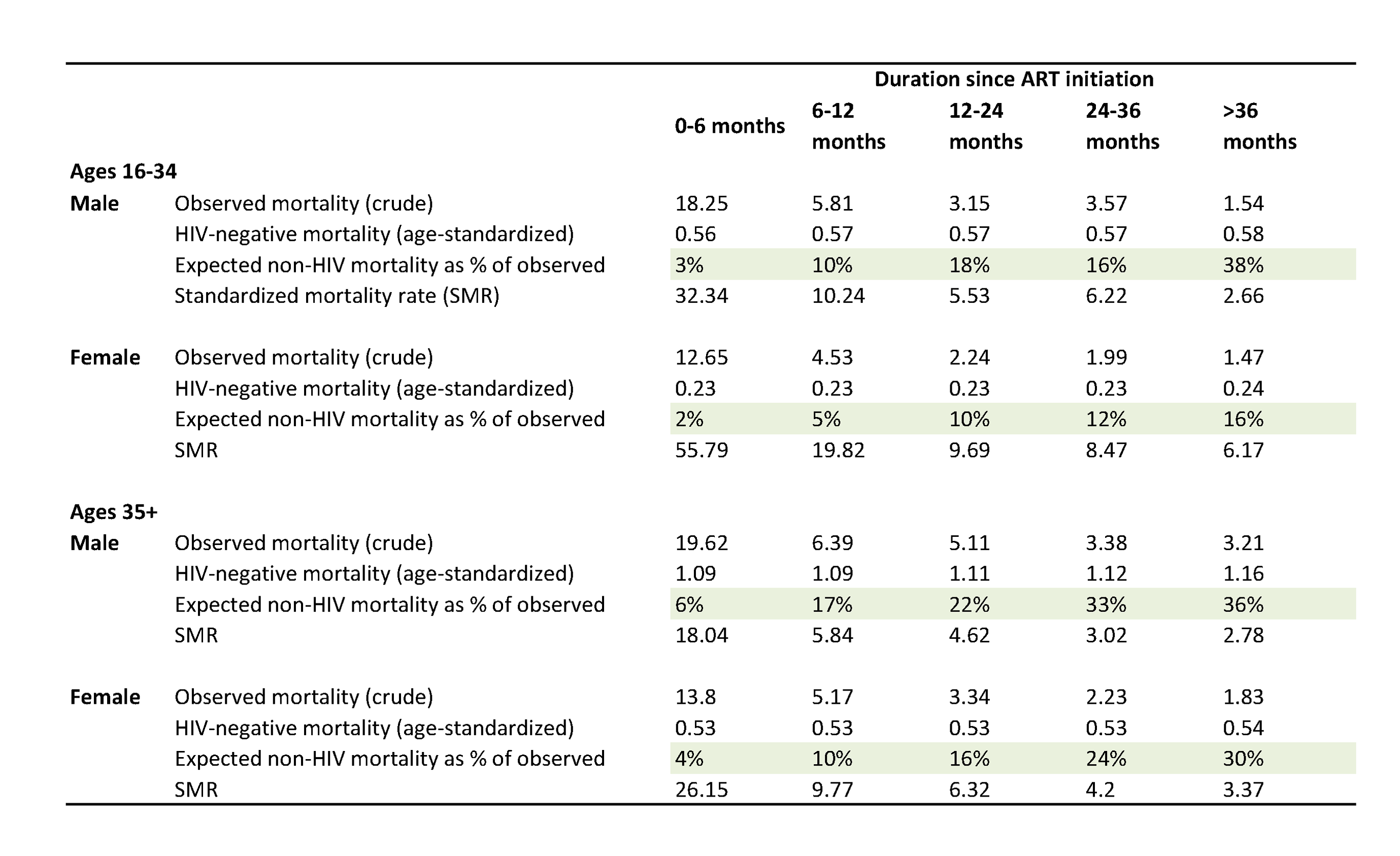

Supplement: Table S2 — Mortality by duration on ART, stratified by age. (TIF) [file pmed.1001304.s004.tif]
