# Supplementary material for: Gender Differences in Survival among Adult Patients Starting Antiretroviral Therapy in South Africa: A Multicentre Cohort Study
Source: PLoS Med. 2012 Sep 4;9(9):e1001304. doi: 10.1371/journal.pmed.1001304 (PMC3433409; doi:10.1371/journal.pmed.1001304)
Supplement: Text S1 — Method for estimating non-HIV mortality. (DOCX) [file pmed.1001304.s005.docx]

**Text S1:**

**Method for estimating non-HIV mortality**

Due to the lack of vital registration systems in many developing countries,[[31](#_ENREF_31)] direct estimates of mortality by age and sex are often not available. To address this problem, life tables are often estimated for developing countries by taking ‘standard’ life tables (which define mortality rates by individual age and sex) and adjusting these to be consistent with aggregate measures of mortality (such as the under-5 mortality rate or probability of death between ages 15 and 60), as estimated in local surveys.[[32](#_ENREF_32)] For example, in the Brass logit life table system,[[33](#_ENREF_33)] the proportion of individuals who survive to age *x* (*l_x_*) is modelled using the equation

$\frac{1}{2}\ln\left(\frac{1-l_{x}}{l_{x}}\right)=\alpha+\frac{1}{2}\beta\ln\left(\frac{1-l_{x}^{S}}{l_{x}^{S}}\right)$,

where $l_{x}^{S}$ is the proportion of individuals who survive to age *x* in the standard life table. In this model there are only two parameters that need to be estimated in adjusting the standard life table to the local population: *α* and *β* (the former determines the adjustment to the overall level of mortality, the latter determines the adjustment to the age gradient in mortality rates).

In South Africa and other countries facing generalized HIV/AIDS epidemics, the use of these simple adjustments to standard life tables has become problematic because of the dramatic change in the patterns of mortality (by age and sex) caused by AIDS. One possible solution to this problem is to split all-cause mortality into HIV mortality and non-HIV mortality, the former being estimated by mathematical models that are fitted to local HIV prevalence data, and the latter being estimated through the adjustment of standard life tables (on the assumption that AIDS would not substantially change the age pattern of non-HIV mortality). This is the process that has been followed in the estimation of mortality rates in the ASSA2008 AIDS and Demographic model, published by the Actuarial Society of South Africa (ASSA).[[34](#_ENREF_34)] The procedure followed is to

1. use estimates of mortality in 1985 (when HIV prevalence in the South African population was negligible[[35](#_ENREF_35),[36](#_ENREF_36)]) as the standard in the estimation of non-HIV mortality;
2. use the time-dependent Brass logit life table approach to define non-HIV mortality in each subsequent year;
3. use ASSA2008 estimates of HIV-related mortality in each year (based on calibration to South African HIV prevalence data from antenatal clinic surveys and household surveys); and
4. vary the *α* and *β* parameters and model HIV parameters until the combined model estimates of all-cause mortality (by age and sex) are consistent with the numbers of deaths in each year, as recorded in the National Population Register,[[37](#_ENREF_37)] after correcting for under-reporting of deaths.

An obvious limitation of this method is that it involves the estimation of non-HIV mortality without any direct data on the cause of death. However, it is important to note that because of the very distinct change in the pattern of deaths by age and sex, brought about by AIDS, the age- and sex-specific death data over time are actually very informative regarding the extent of AIDS mortality, even without cause-of-death data. It is also worth noting that an independent assessment, based on cause-of-death data, has validated the estimates of AIDS mortality produced by a previous version of the ASSA model.[[38](#_ENREF_38)] Although the non-AIDS mortality estimates cannot be quantified with pinpoint accuracy, they are likely to be roughly of the right order of magnitude.

In the present analysis, we have used the ASSA2008 lite model estimates of non-HIV mortality in 2005. Although other years could have been chosen, 2005 was selected because it was close to the median date of ART enrolment in the IeDEA-SA collaboration,[[39](#_ENREF_39)] and because ART coverage in South Africa in 2005 was still relatively low[[40](#_ENREF_40)] (so that potential bias due to mis-specification of mortality on ART would not have been substantial). The ASSA2008 estimates of non-HIV mortality do not change substantially by year; for example, annual non-HIV mortality probabilities in 40-year old men drop steadily from 0.0079 in 2000 to 0.0074 in 2007, and corresponding probabilities in 40-year old women drop from 0.0041 in 2000 to 0.0037 in 2007. Estimates of non-HIV mortality would therefore not change substantially if alternative years were selected.

**References**

1. Setel PW, Macfarlane SB, Szreter S, Mikkelsen L, Jha P, Stout S, et al. A scandal of invisibility: making everyone count by counting everyone. *Lancet*. 2007;370(9598):1569-77.

2. Murray CJL, Ferguson BD, Lopez AD, Guillot M, Salomon JA, Ahmad O. Modified logit life table system: principles, empirical validation, and application. *Pop Studies*. 2003;57(2):165-82.

3. Brass W. On the scale of mortality. In: Brass W, editor. Biological Aspects of Demography. London: Taylor & Francis; 1971.

4. Actuarial Society of South Africa. *ASSA2008 AIDS and Demographic Model*; 2011. Available from: <http://aids.actuarialsociety.org.za>. Accessed 5 April 2011.

5. Dusheiko GM, Brink BA, Conradie JD, Marimuthu T, Sher R. Regional prevalence of hepatitis B, delta, and human immunodeficiency virus infection in southern Africa: a large population survey. *Am J Epidemiol*. 1989;129(1):138-45.

6. Schoub BD, Lyons SF, McGillivray GM, Smith AN, Johnson S, Fisher EL. Absence of HIV infection in prostitutes and women attending sexually-transmitted disease clinics in South Africa. *Trans R Soc Trop Med Hyg*. 1987;81(5):874-5.

7. Statistics South Africa. *Mortality and causes of death in South Africa, 2008: Findings from death notification*. Pretoria; 2010. Available from: <http://www.statssa.gov.za/publications/statsdownload.asp?PPN=P0309.3&SCH=4790>. Accessed 28 Nov 2010.

8. Groenewald P, Nannan N, Bourne D, Laubscher R, Bradshaw D. Identifying deaths from AIDS in South Africa. *AIDS*. 2005;19(2):193-201.

9. Cornell M, Technau K, Fairall L, Wood R, Moultrie H, van Cutsem G, et al. Monitoring the South African antiretroviral programme 2003-2007: the IeDEA Southern Africa Collaboration. *S Afr Med J*. 2009;99(9):653-60.

10. Adam MA, Johnson LF. Estimation of adult antiretroviral treatment coverage in South Africa. *S Afr Med J*. 2009;99(9):661-7.
